# Increased risk of dental trauma in patients with allergic rhinitis: A nationwide population-based cohort study

**DOI:** 10.1371/journal.pone.0182370

**Published:** 2017-07-31

**Authors:** Ming-Jhih Siao, Gunng-Shinng Chen, Wei-Cheng Lee, Jorng-Tzong Horng, Cheng-Wei Chang, Chung-Hsing Li

**Affiliations:** 1 Division of Orthodontics & Dentofacial Orthopedics and Pediatric Dentistry, Department of Dentistry, Tri-Service General Hospital, Taipei, Taiwan; 2 School of Dentistry & Graduate Institute of Dental Science, National Defense Medical Center, Taipei, Taiwan; 3 Department of Computer Science and Information Engineering, National Central University, Chungli, Taiwan; 4 Department of Biomedical Informatics, Asia University, Taichung, Taiwan; 5 Department of Information Management, Hsing Wu University, New Taipei City, Taiwan; Beijing Tongren Hospital, CHINA

## Abstract

Allergic rhinitis (AR) is associated with various developmental issues that affecting dentition. We aimed to determine whether AR is associated with an increased risk of traumatic dental injuries (TDIs) in Taiwanese individuals. We used the Taiwan National Health Insurance Research Database (NHIRD) to conduct a nested case-control study. We compared an AR cohort with a matched cohort of patients without AR. New TDI cases were determined during our study period. To compare TDI risk between our study cohorts, we used Cox proportional regression analysis, and hazard ratios (HR) with 95% confidence intervals (CI) were calculated to quantify the association between AR exposure and TDI risk. In total, 76749 patients with AR (31715 male; 45034 female) were identified. In the AR and the non-AR cohorts, 312 patients in total had TDI. Patients with AR had a significantly higher risk of TDI than those without AR (aHR = 1.92; 95% CI = 1.459–2.525; P < 0.001). The risk of TDI was markedly higher in the AR cohort, except in the 3–12-year-old group, and with a CCI ≥ 1. AR patients had a future risk of TDI, indicating a potentially linked disease pathophysiology. The association between AR and TDI is greater among general patients. Clinicians and caregivers should be aware of potential TDI co-morbidity in patients with AR.

## Introduction

Allergic rhinitis (AR) is the most common airway-obstructing disease, affecting 17.9%–26.3% of adolescents [1,2]. This chronic airway pathology is associated with several symptoms: absent nasal airflow and sneezing, snoring, possible obstructive sleep apnea syndrome, and increased respiratory infections [3,4]. Additionally, it has been reported to increase the risks of inattention and hyperactivity in children [5]. Other studies have also investigated the effect of AR and/or mouth breathing on the general development of cranial-complex [6] and the association with development of dental malocclusion [7–13], including an anterior open bite and a large overjet [14].

This raises the question of whether AR may lead to traumatic dental injury (TDI). TDI is a serious dental public health problem that results in fractured, displaced, or lost teeth and can have significant negative functional, esthetic, and psychological effects. In addition to pain and increased infection risk, the consequences of TDI include changes in physical appearance and speech defects, and can thus affect the patient’s quality of life. TDI is more time-consuming and costly to treat than many other outpatient accidental injuries [15].

**Fig 1:**
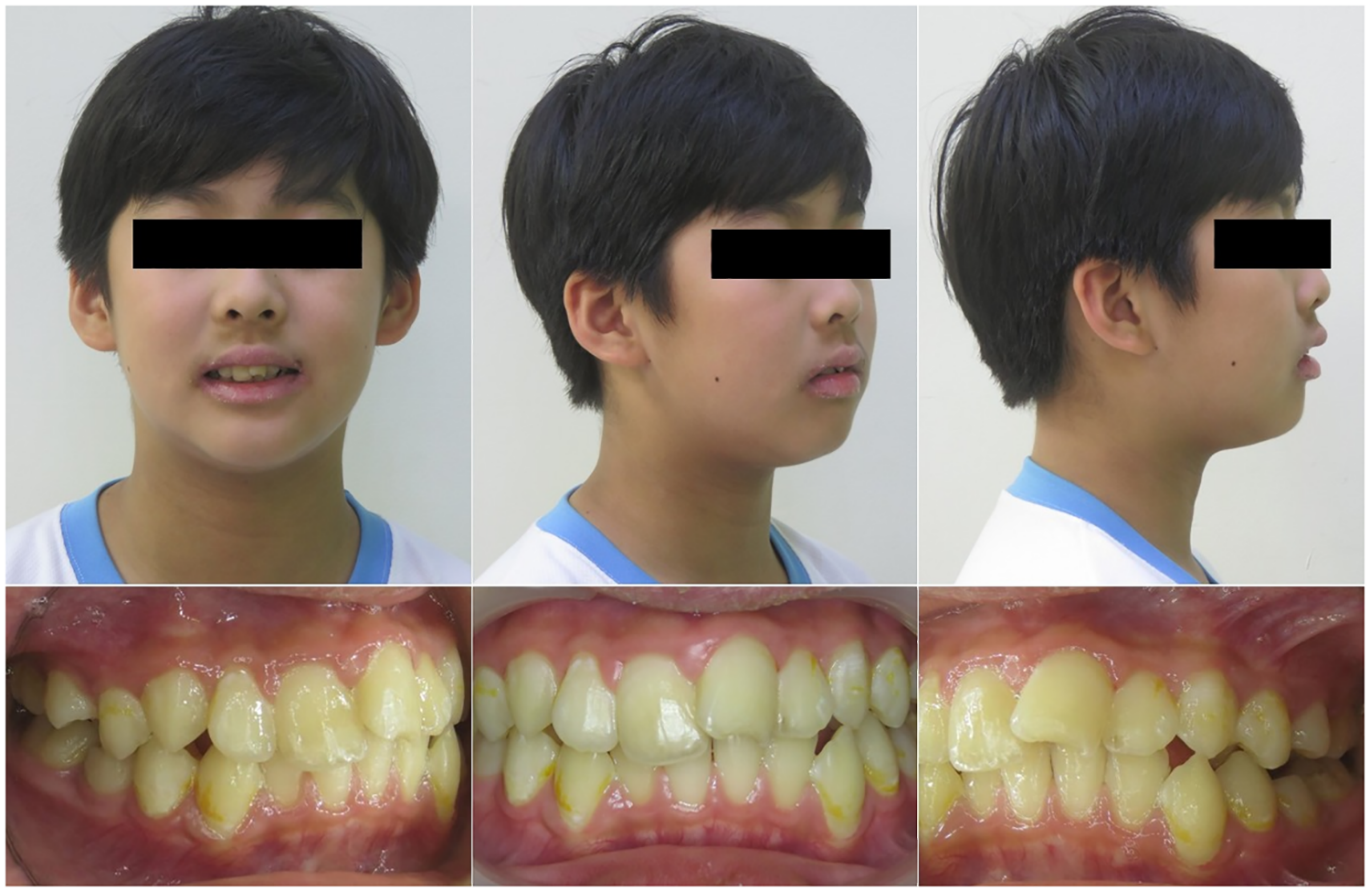
A 13-year-old boy with a history of allergic rhinitis (AR) suffered dental trauma injury at the left maxillary incisor due to sport accident. **The profile shows lip incompetence**, **a weak lip**, **and an acute nasal labial angle**. **The oral finding was Angle class II malocclusion with a large overjet and proclinating incisors**. Consent to publication was obtained form the parents of the patient (S1 File.)

TDI occurs frequently in children and young adults, comprising 5% of all injuries. Boys are generally injured more frequently than girls [16]. Generally, the most frequently affected teeth are the maxillary central incisors (93.3%) [17,18]. These teeth usually protrude and may have inadequate lip coverage [19,20]. The Angle class II, division 1 malocclusion has also been found to be more prone to dental trauma [21] (Fig 1). The etiologies of TDI comprise a broad spectrum of variables, including oral and environmental factors and human behavior [22]. Nevertheless, very few studies have focused on the association between AR and TDI prevalence.

Our study aimed to determine the risk of TDI in patients with AR in Taiwan. We conducted a retrospective cohort study to estimate the relative risk of the incidence of TDI in a nationwide AR cohort, as compared with a non-AR cohort selected from a 1 million representative population. We hypothesized that we would be able to predict the tendency for TDI in AR patients. In the high-risk group, more attention was given to prevention of TDI by preventative and interceptive orthodontic treatment and by providing a safe environment. Furthermore, these findings may be used to influence public policies in long-term care and childcare.

## Methods

### Study design

This study employed a retrospective cohort study design.

### Data source

The Taiwan National Health Insurance Program was established in 1995. This system provides universal health coverage and equal medical access to all Taiwanese individuals. In 2011, the coverage rate of the National Health Insurance was 99.6%; almost the entire population of Taiwan (23 million) was enrolled in this program. A computerized database (Taiwan National Health Insurance Research Database, NHIRD) was set up, derived from the Taiwan National Health Insurance Program and managed by the Taiwan National Health Research Institute (NHRI), which is a nonprofit foundation established by the government. The NHIRD includes patients’ demographic information, encrypted identification numbers, sex, birth dates, admission dates, diagnostic data and procedures, dates of diagnosis, dates of medical treatment, International Classification of Diseases, Ninth Revision, Clinical Modification (ICD-9-CM) diagnostic codes, and drug codes.

### Study population

We conducted a nested case-control study using the NHIRD. From 2000 to 2008, nationwide cohorts were identified on the basis of diagnostic codes: AR and non-AR codes. The cohorts included in our study included individuals aged > 3 years.

### Ethics statement

The NHIRD encrypts patients’ personal information to protect privacy, and provided researchers with anonymous identification numbers associated with relevant claims information, including the patient’s sex, date of birth, medical services received, and prescriptions. Therefore, patient consent was not required to access the NHIRD [23].

### Data availability statement

All data and related metadata were deposited in an appropriate public repository. The data on the study population obtained from the NHIRD are maintained in the NHIRD (http://nhird.nhri.org.tw/).

### Definition of allergic rhinitis exposure

The AR cohort was enrolled from the nationwide Taiwanese population diagnosed with AR (ICD-9: 477.0, 477.1, 477.2, 477.8, 477.9) in 2000, whereas those diagnosed with AR before that year were excluded. In addition, we excluded patients with any acute conditions, such as those with ICD-9 codes for physician-diagnosed cerebrovascular disease and epilepsy, to reduce possible confounding effects.

For comparison, the non-AR cohort was identified from among individuals aged > 3 years and who had no AR diagnostic codes. Each patient with AR was matched to a non-AR patient on the basis of age, sex, duration of enrollment, and cohort entry date. All patient in our cohorts were followed up until dental trauma occurred or until 2010.

### Outcome measurement

As outcomes, we focused on the risk of dental trauma with AR. During our study period, we determined all physician-diagnosed dental trauma cases. Diagnostic codes for dental trauma included ICD-9:873.63—open wound of tooth (broken) (fractured) (owing to trauma), without mention of complications—and 873.73—open wound of tooth (broken; fractured; owing to trauma), complicated.

### Statistical analysis

Chi-square tests were used to compare the baseline characteristics between our two cohorts. We used Cox proportional regression models to compare the risk of dental trauma and to determine adjusted hazard ratios (aHR), which were adjusted for patients’ age, sex, and their Charlson comorbidity index (CCI) score. The non-AR data were used as a reference. A P-value of < 0.05 was considered statistically significant. The cumulative incidence curves of dental trauma in the study cohort were estimated using Kaplan-Meier analyses and the differences between the two cohorts were compared using the log-rank test. All analyses were performed using SAS software, version 9.2 (SAS Institute, Cary, NC, USA).

## Results

In total, 76749 patients with AR (31715 males; 45034 females) were identified during the period of 2000–2008, as shown in Table 1. The maximum follow-up period was 8 years, and the average follow-up period was about 6 years (AR: 6.00 ± 1.72 years; non-AR: 6.16 ± 1.80 years). The ages in the study cohorts were classified into four groups: primary and mixed dentition (3–12-years-old), early permanent dentition (12–29-years-old), middle permanent dentition (29–50-years-old), and older permanent dentition (≥ 50-years-old).

**Table 1:** Demographic characteristics of patients with allergic rhinitis and those without non-allergic rhinitis.

Descriptor		Allergic Rhinitis			
		Yes		No	
		(n = 76749)		(n = 76749)	
Age (years) mean ± SD (P value = 1.000)		32.74 ± 18.38		32.97 ± 18.07	
Age group (years)	3–12	11458	14.93%	11458	14.93%
	12–29	23585	30.73%	23585	30.73%
	30–49	27804	36.23%	27804	36.23%
	≥ 50	13902	18.11%	13902	18.11%
Sex (P value = 1.000)	Female	31715	41.32%	31715	41.32%
	Male	45034	58.68%	45034	58.68%
CCI score\Mean (P value < 0.0001)		0.46 ± 0.87		0.09 ± 0.42	
CCI Low (0)		53461	69.66%	71969	93.77%
CCI Moderate (1)		16166	21.06%	3309	4.31%
CCI High (≥ 2)		7122	9.28%	1471	1.92%

There were no significant differences between the AR and non-AR cohorts in terms of age, sex, and duration of follow-up, as shown in Table 1. Deyo’s CCI [24] was used for designating systemic disease status into three groups (low, moderate, and high). However, there were statistically significant differences in the CCI score between AR and non-AR cohorts.

**Fig 2:**
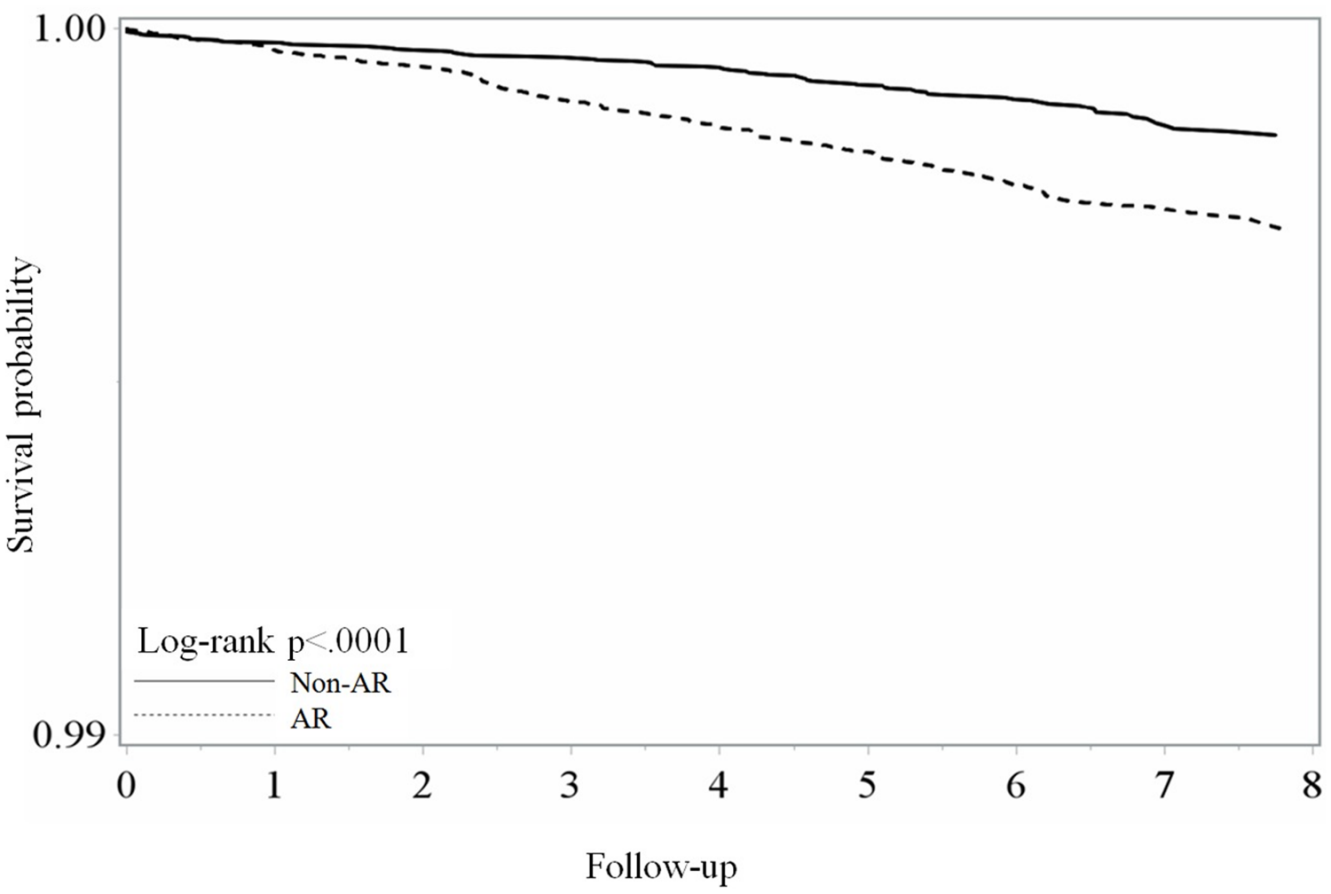
Kaplan-Meier model for estimating dental trauma-free allergic rhinitis patients and non-allergic rhinitis patients.

During our study period, 312 dental trauma cases were identified in the cohorts, involving 218 patients with AR and 94 without AR, as shown in Table 2. After adjusting for age, sex, and CCI score groups, respectively, the risk of dental trauma was higher in the AR (aHR = 1.920; 95% CI = 1.459–2.525; P value < 0.001) than in the non-AR cohort. Both males and females showed clear significance in this respect (aHR = 1.873 and 1.982, respectively). A positive relationship was seen in the AR cohort as compared to the non-AR cohort in the age groups with early permanent dentition (12–29-years-old), middle permanent dentition (29–50-years-old) and, older permanent dentition (≥ 50-years-old). For the CCI (0) group, which had fewer systemic diseases, displayed a higher risk of TDI in the AR than in the non-AR cohort (aHR = 1.783, P < 0.001). In fact, the Kaplan—Meier model (Fig 2) also showed a higher risk in the AR than in the non-AR cohorts overall.

**Table 2:** Incidence and hazard ratios of dental trauma among patients with allergic rhinitis as compared with those without non-allergic rhinitis, based on demographic characteristics and comorbidity.

Allergic rhinitis		Yes		No		Adjusted HR (95% CI)
Variables		Event	Rate	Event	Rate	
All		218	0.47	94	0.20	1.920 ***(1.459–2.525)
Sex	Female	90	0.45	37	0.18	1.982** (1.291–3.042)
	Male	128	0.49	57	0.21	1.873** (1.310–2.276)
Age	3–12	25	0.32	11	0.14	1.612 (0.704–3.689)
	12–29	57	0.40	30	0.20	1.721*(1.045–2.832)
	30–49	70	0.44	37	0.23	1.626* (1.043–2.534)
	≥ 50	66	0.81	16	0.19	3.333** (1.745–6.369)
CCI score \ Mean						
CCI Low (0)		135	0.42	88	0.20	1.783 ***(1.323–2.404)
CCI Moderate (1)		54	0.56	6	0.27	1.702 (0.708–4.096)
CCI High (≥ 2)		29	0.73	0	0.00	5333978 (0.000)

* *P* <0.05;

** *P* <0.01;

*** *P* < 0.001

## Discussion

TDI is a condition with a poor prognosis that influences appearance and function. Our aim was to estimate the risk of TDI in patients with AR, to determine the need for further preventive treatment and for measures to make the environment safe. The database used in this study was released for research purposes by the National Health Research Institutes in 2008. As of 2007, 98.4% of Taiwan’s population (approximately 22.96 million) was enrolled in the NHIRD. The data of the present study were retrieved from 1 million randomly sampled enrollees from the total NHIRD. In this nationwide, population-based, randomized, and longitudinal study, we demonstrated an increased risk of TDI in AR patients, which was 1.92-fold higher than that in the non-AR cohort, after adjustment for sex, age, and medical comorbidities. In each group, the risk of TDI was markedly higher in the AR cohort, except in the 3–12-year-old group, and with a CCI ≥ 1. In the CCI (0) group in particular, excluding most systemic diseases would be more near really risk of TDI with AR. These results support our hypothesis that patients with AR have a higher prevalence of TDI,

Airway obstruction is the main syndrome of AR, even if it is not a continuous phenomenon. AR is considered to be one of the major causes of airway obstruction in children [25], which can induce a loss of habitual physiological nasal breathing. This consequently alters the child’s growth pattern, causing changes in the adenoids [26], long-face syndrome [27], and respiratory obstruction syndrome [28] have been reported, which affect craniofacial development. On one hand, these diseases are expected to result in reduced hypotonicity of the upper and lower lips [6,29], increased incisor proclination [30], anterior open bite, and Class II malocclusion [15]. On the other hand, the oral predisposing factors of TDI are inadequate lip coverage and increased overjet, with dental protrusion [31–37]. Consequently, the characteristics of soft tissue and malocclusion may be the reason for the increased risk of TDI in patients with AR.

In our study, we excluded patients with cerebrovascular disease and epilepsy to avoid the factors of iatrogenic injury and illness. However, the risk of TDI may be affected by the presence of other systemic diseases that cause oral complications, such as periodontitis or trismus. Deyo’s CCI score differed significantly between the AR and non-AR cohorts, with the AR cohort having more systemic diseases. This tendency may elevate the risk of TDI. To identify the health levels in patients with AR, we used the CCI to discriminate between patients with AR and those without AR, in 3 groups: low (CCI = 0), moderate (CCI = 1) and high (CCI ≥ 2). We found statistically significant differences between cohorts in the low CCI group (aHR = 1.783, P-value = 0.0001), but not in the moderate and high CCI groups. The results expressed the risk of TDI between AR and non-AR patients affected by systemic diseases; the CCI = 0 group approximated the risk of TDI between AR and non-AR patients without systemic diseases.

In the various age groups, the primary and mixed dentition group did not attain statistically significant differences between the groups (Table 2). In the primary and mixed dentition group, dental arch development continued to progress; unstable occlusion and tooth position may confound the influence of AR. After maturation of permanent dentition, the effect of AR persisted statistically significantly in the early, middle, and older permanent dentition groups. Moreover, in the older permanent dentition group, the risk of TDI was markedly elevated in the AR cohort (3.3-fold higher than that in the non-AR cohort), which may be because the dentition keeps changing over time, particularly in older patients, as the tissue supporting the teeth is lost. The change in older patients with AR and the accompanying features (mouth breathing and tongue thrusting) may accelerate formation of Class II malocclusion and may increase the TDI risk [38].

AR also affects human behavioral disorders, such as ADHD and oppositional defiant disorder, particularly among children [39]. Nevertheless, in our study, the early and middle permanent dentition groups had similar results, but the differences were not significant between the primary and mixed dentition groups (3–12-years-old). This may be because the environment plays a more important role than human behavior [22]. The prevalence of TDI in children may depend more on caregivers, family, and school.

The study had the following limitations. First, the NHIRD did not include orthodontic and cosmetic patients; such corrections may reduce the developmental disorder caused by AR. Second, the maximal follow-up time of our study was 8 years; however, developmental disorders caused by AR persist life-long. Although there were clear differences in the follow-up time between patients with AR and those without AR (Fig 2), further long-term follow-up studies are required.

In conclusion, this large-scale, nationwide, population-based, longitudinal study demonstrated an association between AR and the risk of TDI. Although the exact mechanism is presently unclear, these findings may provide further insights into the association and possible shared pathophysiology between AR and TDI. The importance of prevention measures for TDI in patients with AR should be emphasized by ensuring a safer environment. Furthermore, these findings may be used to influence public policies in long-term care and childcare.

## Supporting information

S1 File Patient consent form. (PDF)

## References

[1] Guerra S, Sherrill DL, Baldacci S, Carrozzi L, Pistelli F, Di Pede F, et al Rhinitis is an independent risk factor for developing cough apart from colds among adults. Allergy 2005;60: 343–349. doi: 10.1111/j.1398-9995.2005.00717.x 15679720 10.1111/j.1398-9995.2005.00717.x

[2] Hwang CY, Chen YJ, Lin MW, Chen TJ, Chu SY, Chen CC, et al Prevalence of atopic dermatitis, allergic rhinitis and asthma in Taiwan: a national study 2000 to 2007. Acta Derm Venereol. 2010;90: 589–594. doi: 10.2340/00015555-0963 21057741 10.2340/00015555-0963

[3] Skoner DP. Allergic rhinitis: definition, epidemiology, pathophysiology, detection, and diagnosis. J Allergy Clin Immunol. 2001;108(1 Suppl.): S2–S8. 11449200 10.1067/mai.2001.115569

[4] Bellanti JA, Wallerstedt DB. Allergic rhinitis update: epidemiology and natural history. Allergy Asthma Proc. 2000;21: 367–370. 11191103 10.2500/108854100778249088

[5] Yang MT, Lee WT, Liang JS, Lin YJ, Fu WM, Chen CC. Hyperactivity and impulsivity in children with untreated allergic rhinitis: corroborated by rating scale and continuous performance test. Pediatr Neonatol. 2014;55: 168–174. doi: 10.1016/j.pedneo.2013.09.003 24211085 10.1016/j.pedneo.2013.09.003

[6] Valera FC, Travitzki LV, Mattar SE, Matsumoto MA, Elias AM, Anselmo-Lima WT. Muscular, functional and orthodontic changes in pre-school children with enlarged adenoids and tonsils. Int J Pediatr Otorhinolaryngol. 2003;67: 761–770. 12791452 10.1016/s0165-5876(03)00095-8

[7] Cheng MC, Enlow DH, Papsidero M, Broadbent BH Jr, Oyen O, Sabet M. Developmental effects of impaired breathing in the face of the growing child. Angle Orthod. 1988;58: 309–320. 3207212 10.1043/0003-3219(1988)058<0309:DEOIBI>2.0.CO;2

[8] Principato JJ. Crossbite and increased anterior vertical height secondary to oral respiration. Proceedings of the 6th International College of Cranio-Mandibular. Orthopedics. Florence, Italy. 1989; April 22–23.

[9] Freng A. Restricted nasal respiration, influence on facial growth. Int J Paediatr Otorhinolaryngol. 1979;1: 249–254. 10.1016/0165-5876(79)90019-3 552382

[10] Vázquez Nava F, Vázquez Rodríguez EM, Reyes Guevara S, Barrientos Gómez Mdel C, Vázquez Rodriguez CF, Saldivar González AH, et al Effect of allergic rhinitis, asthma and rhinobronchitis on dental malocclusion in adolescents. Rev Alerg Mex. 2007;54: 169–176. 18693539

[11] Bresolin D, Shapiro PA, Shapiro GG, Chapko MK, Dassel S. Mouth breathing in allergic children: its relationship to dentofacial development. Am J Orthod. 1983;83: 334–340. 6573147 10.1016/0002-9416(83)90229-4

[12] Hartgernik DV, Vig PS. Lower anterior face height and lip incompetence do not predict nasal airway obstruction. Angle Orthod. 1989; 59: 17–23. 2646989 10.1043/0003-3219(1989)059<0017:LAFHAL>2.0.CO;2

[13] Souki BQ, Pimenta GB, Souki MQ, Franco LP, Becker HM, Pinto JA. Prevalence of malocclusion among mouth breathing children: do expectations meet reality? Int J Pediatr Otorhinolaryngol. 2009;73: 767–773. doi: 10.1016/j.ijporl.2009.02.006 19282036 10.1016/j.ijporl.2009.02.006

[14] Vázquez Nava F, Vázquez Rodríguez EM, Reyes Guevara S, Barrientos Gómez Mdel C, Vázquez Rodriguez CF, Saldivar González AH, et al Effect of allergic rhinitis, asthma and rhinobronchitis on dental malocclusion in adolescents. Revista Alergia México 2007;54: 169–176. 18693539

[15] Agostinho HA, Furtado IÃ, Silva FS, Ustrell Torrent J. Cephalometric evaluation of children with allergic rhinitis and mouth breathing. Acta Med Portuguesa 2015;28: 316–321. 10.20344/amp.5556 26421783

[16] Andersson L. Epidemiology of traumatic dental injuries. J Endodont. 2013;39: S2–S5. 10.1016/j.joen.2012.11.021 23439040

[17] Kowash MB, Fayle SA, Curzon ME. A retrospective analysis of traumatic injuries to permanent incisor teeth. Ital J Ped Dent.1999;1: 25–30.

[18] Calskan MK, Turkun M. Clinical investigation of traumatic injuries of permanent incisors in Izmir, Turkey. Endod Dent Traumatol.1995;11: 210–213. 8625933 10.1111/j.1600-9657.1995.tb00490.x

[19] Cortes MI, Marcenes W, Sheiham A. Prevalence and correlates, of traumatic injuries to the permanent teeth of school-children aged 9–14 years in Belo Horizonte, Brazil. Dent Traumatol.2001;17: 22–26. 11475767 10.1034/j.1600-9657.2001.170105.x

[20] Marcenes W, Alessi ON, Traebert J. Causes and prevalence of traumatic injuries to the permanent incisors of school children aged 12 years in Jaragua do Sul, Brazil. Int Dent J. 2000;50: 87–92. 10945187 10.1002/j.1875-595x.2000.tb00804.x

[21] Dua R, Sharma S. Prevalence, causes, and correlates of traumatic dental injuries among seven-to-twelve-year-old school children in Derabassi. Contemp Clin Dent. 2012;3: 38–41. doi: 10.4103/0976-237X.94544 22557895 10.4103/0976-237X.94544 PMC3341757

[22] Glendor ULF. Aetiology and risk factors related to traumatic dental injuries—a review of the literature. Dent Traumatol. 2009;25:19–31. doi: 10.1111/j.1600-9657.2008.00694.x 19208007 10.1111/j.1600-9657.2008.00694.x

[23] Yeh Jun-Jun, Wang YC, Chen JH, Hsu WH. Effect of systemic lupus erythematosus on the risk of incident respiratory failure: A national cohort study. PloS One, 2016, 11 9: e0163382 doi: 10.1371/journal.pone.0163382 27654828 10.1371/journal.pone.0163382 PMC5031430

[24] Deyo RA, Cherkin DC, Ciol MA. Adapting a clinical comorbidity index for use with ICD-9-CM administrative databases. J Clin Epidemiol. 1992;45: 613–619. 1607900 10.1016/0895-4356(92)90133-8

[25] Abreu RR, Rocha RL, Lamounier JA, Guerra AF. Etiology, clinical manifestations and concurrent findings in mouth-breathing children. J Pediatr. 2008;84: 529–535. 10.2223/JPED.1844 19060979

[26] Rubin RM. Mode of respiration and facial growth. Am J Orthodont. 1980;78: 504–510. 6933857 10.1016/0002-9416(80)90301-2

[27] Schendel SA, Eisenfeld J, Bell WH, Epker BN, Mishelevich CJ. The long face syndrome: vertical maxillary excess. Am J Orthod. 1976;70: 398–408. 1067758 10.1016/0002-9416(76)90112-3

[28] Ricketts RM. Respiratory obstruction syndrome. Am J Orthod. 1968;54: 495–414. 5240645 10.1016/0002-9416(68)90218-2

[29] Basheer B, Hegde KS, Bhat SS, Umar D, Baroudi K. Influence of mouth breathing on the dentofacial growth of children: a cephalometric study. J Int Oral Health 2014;6: 50–55. PMC4295456 25628484

[30] Frasson JM, Magnani MB, Nouer DF, de Siqueira VC, Lunardi N. Comparative cephalometric study between nasal and predominantly mouth breathers. Braz J Otorhinolaryngol. 2006;72: 72–81. 16917556 10.1016/S1808-8694(15)30037-9 PMC9445764

[31] Burden DJ. An investigation of the association between overjet size, lip coverage, and traumatic injury to maxillary incisors. Eur J Orthod. 1995;17: 513–517. 8682168 10.1093/ejo/17.6.513

[32] Marcenes W, Al Beiruti N, Tayfour D, Issa S. Epidemiology of traumatic injuries to the permanent incisors of 9–12-year-old schoolchildren in Damascus, Syria. Endod Dent Traumatol.1999;15: 117–123. 10530154 10.1111/j.1600-9657.1999.tb00767.x

[33] Bauss O, Rohling J, Schwestka-Polly R. Prevalence of traumatic injuries to the permanent incisors in candidates for orthodontic treatment. Dent Traumatol. 2004;20: 61–66. 15025687 10.1111/j.1600-4469.2004.00230.x

[34] Årtun J, Behbehani F, Al-Jame B, Kerosuo H. Incisor trauma in an adolescent Arab population: prevalence, severity, and occlusal risk factors. Am J Orthod Dentofacial Orthop. 2005;128: 347–452. 16168331 10.1016/j.ajodo.2004.06.032

[35] Sgan-Cohen HD, Megnagi G, Jacobi Y. Dental trauma and its association with anatomic, behavioural, and social variables among fifth and sixth grade schoolchildren in Jerusalem. Commun Dent Oral Epidemiol. 2005;33: 174–180. 10.1111/j.1600-0528.2005.00202.x 15853840

[36] Traebert J, Bittencourt DD, Peres KG, Peres MA, De Lacerda JT, Marcenes W. Aetiology and rates of treatment of traumatic dental injuries among 12-year-old school children in a town in southern Brazil. Dent Traumatol. 2006;22: 173–178. 16872385 10.1111/j.1600-9657.2006.00359.x

[37] Soriano EP, Caldas AF Jr, Carvalho MVD, Amorim Filho HA. Prevalence and risk factors related to traumatic dental injuries in Brazilian school children. Dent Traumatol. 2007;23: 232–240. 17635357 10.1111/j.1600-9657.2005.00426.x

[38] Cho SH, Kim TH, Kim KR, Lee JM, Lee DK, Kim JH, et al Factors for maxillary sinus volume and craniofacial anatomical features in adults with chronic rhinosinusitis. Arch Otolaryngol Head Neck Surg. 2010;136: 610–615. doi: 10.1001/archoto.2010.75 20566913 10.1001/archoto.2010.75

[39] Lin YT, Chen YC, Gau SS, Yeh TH, Fan HY, Hwang YY, et al Associations between allergic diseases and attention deficit hyperactivity/oppositional defiant disorders in children. Pediatr Res. 2016;80: 480–485. doi: 10.1038/pr.2016.111 27356086 10.1038/pr.2016.111

